# In silico characterization and structural modeling of bacterial metalloprotease of family M4

**DOI:** 10.1186/s43141-020-00105-y

**Published:** 2021-02-02

**Authors:** Rajnee Hasan, Md. Nazmul Haq Rony, Rasel Ahmed

**Affiliations:** Basic and Applied Research on Jute Project, Bangladesh Jute Research Institute, Manik Mia Avenue, Dhaka, 1207 Bangladesh

**Keywords:** M4 family metalloprotease, Bacteria, Structural and functional analysis, Phylogenetic tree, Potential drug targets

## Abstract

**Background:**

The M4 family of metalloproteases is comprised of a large number of zinc-containing metalloproteases. A large number of these enzymes are important virulence factors of pathogenic bacteria and therefore potential drug targets. Whereas some enzymes have potential for biotechnological applications, the M4 family of metalloproteases is known almost exclusively from bacteria. The aim of the study was to identify the structure and properties of M4 metalloprotease proteins.

**Results:**

A total of 31 protein sequences of M4 metalloprotease retrieved from UniProt representing different species of bacteria have been characterized for various physiochemical propertie**s.** They were thermostable, hydrophillic protein of a molecular mass ranging from 38 to 66 KDa. Correlation on the basis of both enzymes and respective genes has also been studied by phylogenetic tree. *B*. *cereus* M4 metalloprotease (PDB ID: 1NPC) was selected as a representative species for secondary and tertiary structures among the M4 metalloprotease proteins. The secondary structure displaying 11 helices (H1-H11) is involved in 15 helix-helix interactions, while 4 β-sheet motifs composed of 15 β-strands in PDBsum. Possible disulfide bridges were absent in most of the cases. The tertiary structure of *B*. *cereus* M4 metalloprotease was validated by QMEAN4 and SAVES server (Ramachandran plot, verify 3D, and ERRAT) which proved the stability, reliability, and consistency of the tertiary structure of the protein. Functional analysis was done in terms of membrane protein topology, disease-causing region prediction, proteolytic cleavage sites prediction, and network generation. Transmembrane helix prediction showed absence of transmembrane helix in protein. Protein-protein interaction networks demonstrated that bacillolysin of *B*. *cereus* interacted with ten other proteins in a high confidence score. Five disorder regions were identified. Active sites analysis showed the zinc-binding residues—His-143, His-147, and Glu-167, with Glu-144 acting as the catalytic residues.

**Conclusion:**

Moreover, this theoretical overview will help researchers to get a details idea about the protein structure and it may also help to design enzymes with desirable characteristics for exploiting them at industrial level or potential drug targets.

**Supplementary Information:**

The online version contains supplementary material available at 10.1186/s43141-020-00105-y.

## Background

Proteases are enzymes that can hydrolyze proteins and are composed of a diverse group of exoproteases and endoproteases depending on their activity. Based on their catalytic mechanism, endoproteases are divided into aspartic proteases, cysteine proteases, metalloproteases, serine proteases, and threonine proteases [1]. Proteases that contain one or two divalent metal ions in their active sites are known as metalloproteases. While most of the metalloproteases contain Zn^2+^, in some cases, Ca^2+^ Mg^2+^, Ni^2+^, or Cu^2+^ are also found [2]. The role of the catalytic metal ions in metalloproteases is to activate the water molecule, which serves as a nucleophile in catalysis. Metalloproteases are produced by all species of plants, animals, and microorganisms. They are involved in many biological processes such as embryonic development, morphogenesis, processing of peptide hormones, release of cytokines and growth factors, cell-cell fusion, cell adhesion and migration, intestinal absorption of nutrients, viral polyprotein processing, bacterial cell wall biosynthesis, and metabolism of antibiotics [3]. Due to their active relation with many diseases, extracellular metalloproteases have been widely studied [4].

All the well-characterized proteinases to date belong to one or more family in MEROPS database (http://MEROPS.sanger.ac.uk/). The current MEROPS database (release 12.1) classifies metallopeptidases into 76 families, which are grouped into sixteen clans based on metal ion binding motifs and similarities to their 3-D structure. The proteases in the M4 family belong to clan MA, a big family of metalloproteases that degrade extracellular proteins and peptides for bacterial nutrition. Metalloproteases of the family M4 comprise different types of peptidases, thermolysin, vibriolysin, pseudolysin, coccolysin, aureolysin, vimelysin, lambda toxin, bacillolysin, stearolysin, gelatinase, elastase, etc.

Some of the metalloproteases are widely used in the food, medicine, brewing, leather, film, and baking industries. Thermolysin from *Bacillus thermoproteolyticus* has diverse industrial usage. Thermolysin is used as a peptide and ester synthetase in the production of N-carbobenzoxy-l-aspartyl-l-phenylalaninemethyl ester (Z-Asp-Phe-OMe), the precursor to the artificial sweetener aspartame [5–7]. Thermolysin is also used in biotechnology industry as a non-specific proteinase to obtain fragments for peptide sequencing. Thermozymes, enzymes from thermophilic microorganisms, have unique characteristics such as extreme temperature persistence, high stability in organic solvents, strict substrate specificity, and pH stability. For these features, thermozymes have been considerably used in many industrial applications [8–11]. Vimelysin from *Vibrio* str.T1800 has pertinence in peptide condensation reactions because of its high activity in organic solvents [12]. In addition, vibriolysin from *Vibrio proteolyticus* are utilized in several industrial as well as biomedical applications [10, 11]. It mediates the coupling of N-protected aspartic acid and phenylalanine methyl ester to yield N-protected aspartylphenylalaninemethylester, a precursor of the sweetener aspartame, whereas a new metalloprotease of the M4 family, VP9, was identified in *Vibrio pomeroyi* strain 12613 from Atlantic seawater that was able to hydrolyze casein and gelatin [13]. Neutrase from *B*. *subtilis* was began to use in industrial sector in 1995 for the synthesis of Celite-545 and followed by 1997 for the synthesis of Polyamide-PA6 [14, 15]. Another metalloprotease of M4 family, pseudolysin from *P*. *aeruginosa*, can be developed for peptide synthesis, which has been demonstrated to be a suitable catalyst for peptide bond formation through reverse proteolysis [16]. M4 metalloprotease obtainable from Actinobacteria is used for wort production [17]. Thus, proteinases of the M4 family have a huge potential for industrial context and have also been found to be a useful catalyst in protein engineering [18, 19].

Members of the M4 family that are considered virulence factors of pathogens can also be used as targets in drug and vaccine development [20, 21]. Lambda toxin of *Clostridium perfringens* activates the precursors of clostridial potent toxins and degrades various host proteins that contribute to innate or adaptive immune defense against infections [22]. Hemagglutinin from *Vibrio cholerae* are the causative agents for gastritis, peptic ulcer, gastric carcinoma [23] and cholera. It has also been shown to affect intracellular tight junctions by degrading occluding [24]. In addition, vibriolysin from *V*. *proteolyticus* is used for the removal of necrotic tissue from wounds such as burns or cutaneous ulcers and is reported to stimulate the healing of partial-thickness burn wounds [25]. The peptidase from *Legionella* may have role in the virulence of Legionnaire’s disease and pneumonia [26] as it cleaves α_1_-antitrypsin [27], tumor necrosis factor α, interleukin 2, and CD4 on human T cell surfaces [28]. Pseudolysin, an extracellullar elastase of *Pseudomonas aeruginosa*, is involved in chronic ulcers by degradation of human wound fluids and human skin proteins [29]. Again, *Pseudomonas aeruginosa* produces elastase B in the hemolymph after infection of larval silkworm contributes to the growth of *P*. *aeruginosa* in the silkworm and pathogenicity of *P*. *aeruginosa* to the host [30]. Based on the current knowledge, it is reasonable to believe that particular metalloproteinases associated with human pathogens have been recognized as prominent virulence factors and their therapeutic inhibition has become a novel strategy in the development of second-generation antibiotics [31, 32].

For a successful integration of M4 metalloprotease in large-scale industrial processes and therapeutic use, a detailed understanding of the enzyme is prerequisite. The present study was aimed to be utilize in silico tools for the characterization of M4 metalloprotease from different bacterial species for their physicochemical characteristics; primary, secondary, and tertiary structure of proteins; functional analysis; domains and motifs; protein model; and phylogenetic analysis.

## Methods

### Sequence retrieval and alignment

A total of 31 different M4 metalloprotease sequences of bacterial origin have been retrieved from UniProt (https://www.uniprot.org/). Corresponding gene sequences of 31 bacterial M4 metalloprotease proteins were retrieved from NCBI (https://www.ncbi.nlm.nih.gov/). The UniProtKB of protein sequences, accession numbers of the gene sequences along with the source organisms were listed in Supplementary Table 1. Clustal Omega (https://www.ebi.ac.uk/Tools/msa/clustalo/) [33] algorithm was used for the alignment of retrieved protein sequences through multiple sequence alignments and the alignments were inspected using CLC sequence viewer 8.0 (http://www.clcbio.com).

### Determination of physical parameters of the proteins

The different physicochemical properties of M4 metalloprotease enzymes were computed using ExPASy’sProtParam tool (http://web.expasy.org/protparam) [34] and these properties were deduced from a protein sequence. The ProtParam includes the following computed parameters: molecular weight, isoelectric point (pI), extinction coefficient (EC—quantitative study of protein-protein and protein-ligand interactions), instability index (II—stability of proteins), aliphatic index (AI—relative volume of protein occupied by aliphatic side chains), and Grand Average of Hydropathicities (GRAVY—sum of all hydropathicity values of all amino acids divided by number of residues in a sequence).

### Phylogenetic tree construction

Two different phylogenetic trees were constructed from amino acid sequences and from gene sequences using the MEGAX software [35] to compare evolutionary relatedness of the taxa. The evolutionary history was inferred using the neighbor-joining method [36]. For amino acid sequence, the evolutionary distances were computed using the Poisson correction method [37] whereas for gene sequence the evolutionary distances were computed using the maximum composite likelihood method [38].

### Primary structure analysis

For primary structure analysis, viz., the amino acids present in polypeptide chain, ExPASy-ProtParam tool had been used. For domain search, the Pfam site (http://www.sanger.ac.uk/Software/Pfam/search.shtml) was used. Motif analysis was done using MEME (http://meme.sdsc.edu/meme/meme.html) [39]. The conserved protein motifs deduced by MEME were subjected to biological functional analysis using protein BLAST and domains were studied with Interproscan (http://www.ebi.ac.uk/interpro/search/sequence/) providing the best possible match based on highest similarity score.

### Secondary structure analysis

Secondary structure analysis of retrieved bacterial M4 metalloproteases included number of α-helices, β-turn, extended strand, β-sheet, and coils which were performed by SOPMA from the Network Protein Sequence Analysis (NPS@) server (https://npsa-prabi.ibcp.fr/cgi-bin/npsa_automat.pl?page=npsa_sopma.html) [34]. The secondary motif map and topology diagram were calculated using the PDBsum tool (http://www.ebi.ac.uk/thornton-srv/databases/cgibin/pdbsum/GetPage.pl?pdbcode=index.html) [40]. The consensus secondary structure contents and predicted disulfide patterns of each protein were tabulated. The presence of disulfide bridges was analyzed using the CYS-REC tool (http://linux1.softberry.com/berry.phtml) which predicted the most probable bonding patterns between available cysteine residues.

### Tertiary structure analysis and validation

Among the 31 strains, *B*. *cereus* was selected as a representative of all the strains to predict the tertiary structure of M4 metalloprotease protein. SWISS-MODEL 3.1.0 (https://swissmodel.expasy.org/) was used to build the 3D models of *B*. *cereus* M4 metalloprotease sequence (PDB ID: 1NPC) with energy minimization parameters [41]. PyMOL (Schrödinger Inc.) was used to visualize publishable image of the model [42]. Structure evaluation was the most important component of structure prediction. Predicted protein model of M4 metalloprotease of *B*. *cereus* was evaluated and verified from both QMEAN (https://swissmodel.expasy.org/qmean/) and SAVES (https://servicesn.mbi.ucla.edu/SAVES/) server. Ramachandran plot generated from RAMPAGE server (http://mordred.bioc.cam.ac.uk/~rapper/rampage.php) [43]. Verify3D [44] and ERRAT [45] were evaluated from SAVES. The overall quality of the structure was obtained through Ramachandran plot. Verify3D analyzed the compatibility of an atomic model (3D) with its own amino acid sequence (1D) [46]. The verification of the crystallographic structure of proteins was done by Errate.

### Functional analysis

Function prediction was done in terms of membrane protein topology, disease-causing region prediction, proteolytic cleavage sites prediction and network generation. TMHMM 2.0 tool (www.cbs.dtu.dk/services/TMHMM) was used to understand membrane protein topology, more specifically if the protein was membrane spanning or extracellular in nature [47]. GlobPlot 2.3 (http://globplot.embl.de/) was used to identify regions of globularity and disorder within protein sequences. This web service looks for order/globularity or disorder tendency in the query protein based on a running sum of the propensity for an amino acid by searching domain databases and sets of disordered proteins [48]. Proteolytic cleavage sites were identified by using a web-based tool peptide cutter (http://web.expasy.org/peptide_cutter/) [34], which predicted the proteolytic cleavage sites and sites cleaved by chemicals in a given protein sequence. Identification of protein-protein interaction was carried out by STRING 11.0 (https://string-db.org/) [49]. STRING is a biological database which is used to construct protein-protein interaction network for different known and predicted protein interactions.

### Active site prediction

The Computed Atlas of Surface Topography of proteins 3.0 (CASTp 3.0) server (http://sts.bioe.uic.edu/) [50] was used to predict active binding site pockets of protein. It includes annotated functional information of specific residues on the protein structure.

## Results

### Sequence retrieval and alignment

**Fig. 1:**
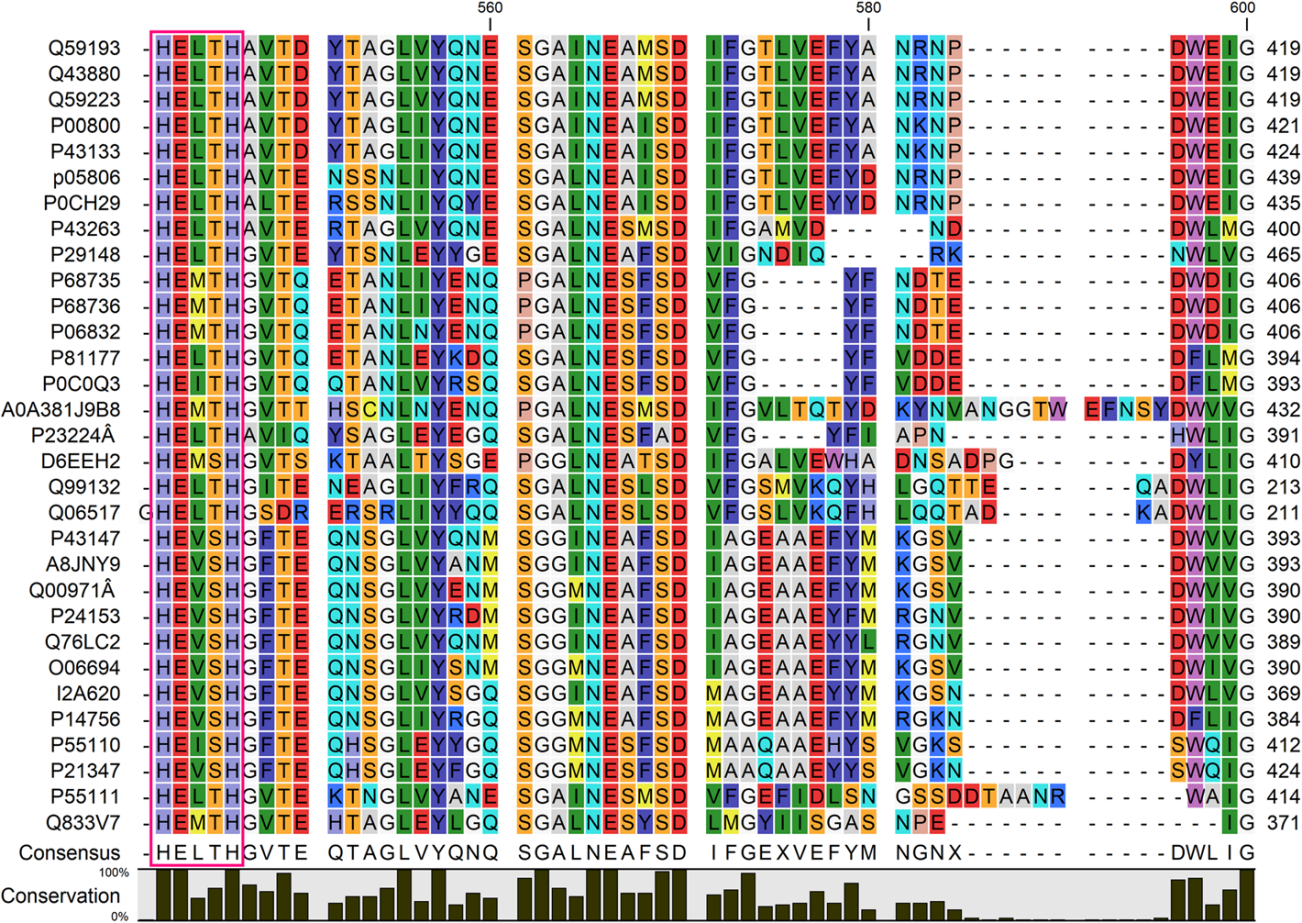
Multiple sequence alignment of M4 metalloprotease amino acid sequences showing the zinc-binding motif “HEXXH” in a pink box

The protein sequences of M4 metalloprotease enzymes belonging to different bacterial strains were retrieved from UniProt and FASTA format of these sequences have been selected based on the overall quality parameters in UniProt tool (Supplementary table 1). The homology search and multiple sequence alignment of these 31 M4 metalloprotease sequences revealed a little stretch of conserved region ranging from the amino acid residues 117–146, 304–323, 451–533, 542–579, 595–624, 652–667, and 705–720 as shown in Figure S1. Few highly conserved amino acids were also observed for most of the sequences. Twenty-two 100% conserved positions were found in aligned region comprising nonpolar amino acid, Ala, Leu, Gly, and Pro, Val; polar amino acid, Asn and Ser; aromatic amino acid, Tyr; acidic amino acid, Glu and Asp; and basic amino acid, Arg and His. Alignment of 31 M4 metalloproteases revealed a conserved region (HELTE) occurring between amino acid positions “542–546” in the region blocked with pink areas of Fig. 1.

### Phylogenetic tree construction

**Fig. 2:**
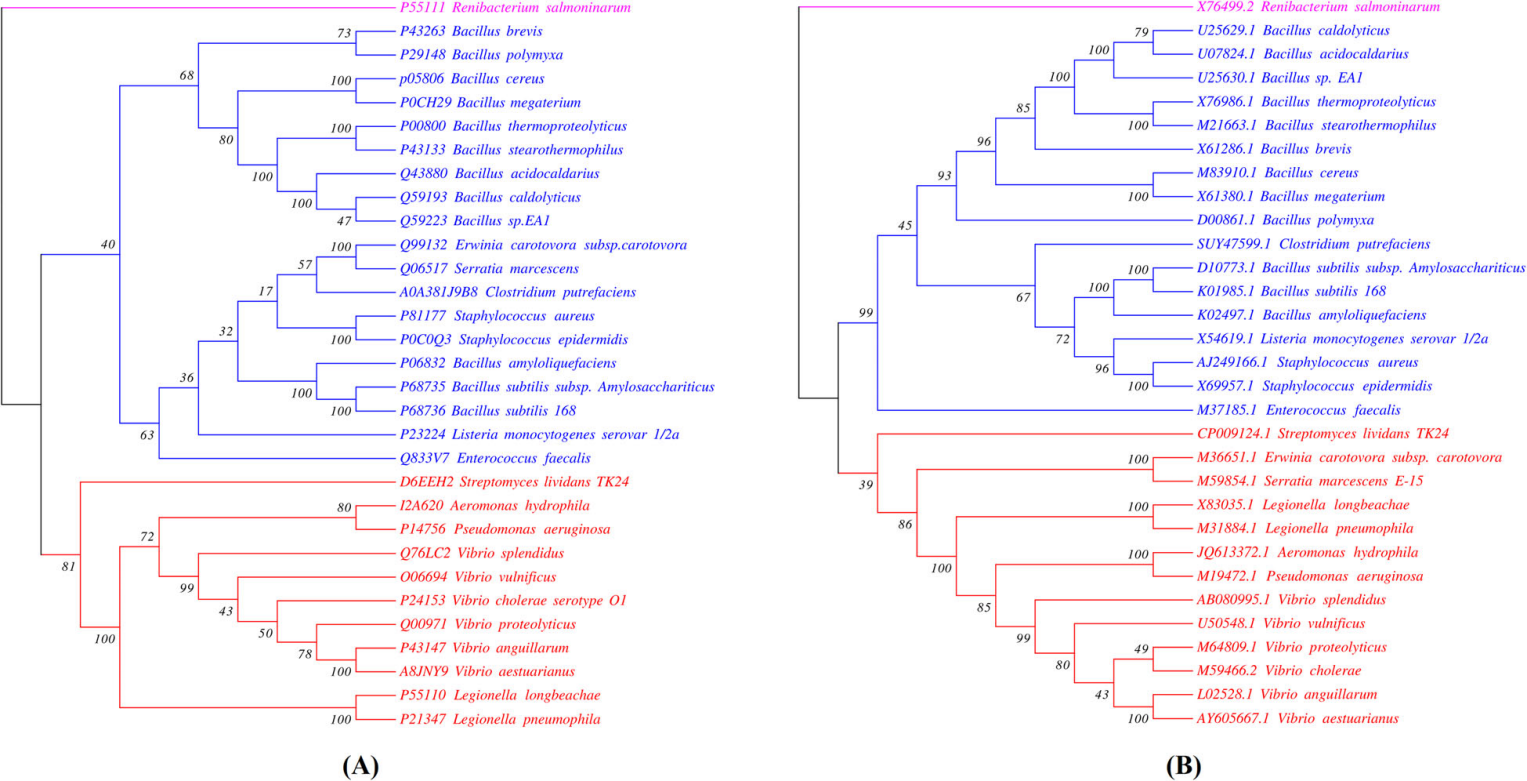
Phylogenetic tree of 31 different M4 metalloproteases of bacterial origin by Neighbor-joining method using MEGA X. **a** Phylogenetic tree constructed with 31 amino acid sequences of bacterial M4 metalloprotease. **b** Phylogenetic tree constructed with 31 gene sequences of bacterial M4 metalloproteases

To compare evolutionary relationship, two phylogenetic trees were constructed with MEGAX, one consisted of amino acid sequences of 31 bacterial M4 metalloprotease enzymes (Fig. 2a), and another one is their corresponding gene sequences (Fig. 2b). The horizontal branches represented evolutionary lineages changing over time. The longer the branch, the larger the amount of change. In Fig. 2a the optimal tree with the sum of branch length = 8.83019656 was shown. Here, amino acid sequences were distributed into two main clades and an outgroup. Dominant clade consisted of amino acid sequences from mostly gram-positive bacteria (except *Streptomyces lividans TK24*; D6EEH2) along with two gram-negative bacteria, *Erwinia_carotovora_subsp*.*_carotovora*; Q99132 and *Serratia marcescens*; Q06517 and were marked as blue (Fig. 2a). From this figure, it was observed that the two strains *Erwinia_carotovora_subsp*.*_carotovora*; Q99132 and *Serratia marcescens*; Q06517 clustered with *Clostridium putrefaciens*; A0A381J9B8 which revealed sequence level similarity of these protein sequences. Whereas amino acid sequences from rest gram-negative bacteria with *Streptomyces lividans TK24*, D6EEH2 exhibited high similarity index and they belonged to another clade and were represented as red (Figure 2a).

In Fig. 2b another phylogenetic tree was constructed to find out the relation among gene sequences of corresponding protein. The optimal tree with the sum of branch length = 20.01342277 was shown. Here, gene sequences of gram-positive bacteria (marked as blue) and gram-negative bacteria (marked as red) formed separate clusters signifying the sequence-based similarity. In both cases, the outgroup contained *Renibacterium salmoninarum* marked as pink.

### Determination of physical parameters of the proteins

In confirmation of the uniqueness of any protein or enzyme molecule, characterization of the biochemical features of these molecules play the preliminary role [34]. The physicochemical features of protease sequences obtaining from ExPASy ProtParam were summarized in Table 1. The total number of amino acid residues ranged from 347 to 611 with variable molecular weights. The pI values of all the proteins showed broad range of 4.88–10. The variability was also observed among these proteins in terms of other physiochemical parameters like negative charge residues (Asp and Glu), positively charged amino acid residues (His, Arg, Lys), hydropathicity (GRAVY), and extinction coefficient (EC) which were listed in Table 1.

**Table 1:** Biochemical features of M4 metalloprotease protein sequences from different bacterial species

S.N.	Bacteria Name	UniProtKB	Seq length	MW	pI	EC	Ii	Ai	GRAVY	−R	+R
1	*Aeromonas hydrophila*	I2A620	590	62969.1	6.02	99615	25.25	69.47	− 0.245	52	46
2	*Bacillus thermoproteolyticus*	P00800	548	60103.9	5.53	81140	26.43	76.35	− 0.392	59	48
3	*B*. *stearothermophilus*	P43133	551	60616.6	5.82	81140	26.78	76.46	− 0.397	59	51
4	*B*. *subtilis Amylosacchariticus*	P68735	521	56521.7	7.16	63720	30.09	74.38	− 0.51	53	53
5	*B*. *subtilis*	P68736	521	56521.7	7.16	63720	30.09	74.38	− 0.51	53	53
6	*B*. *cereus*	P05806	566	60919.3	5.7	75640	29.23	69.98	− 0.484	64	56
7	*B*. *brevis*	P43263	527	58645.7	5.33	90650	29.44	68.67	− 0.598	70	50
8	*B*. *polymyxa*	P29148	590	63528.7	4.88	86640	26.19	75.1	− 0.43	74	50
9	*B*. *caldolyticus*	Q59193	546	59770.6	5.39	102110	29.36	73.97	− 0.372	60	48
10	*B*. *megaterium*	P0CH29	562	60948.6	8.4	83090	29.61	70.64	− 0.52	55	58
11	*B*. *amyloliquefaciens*	P06832	521	56840.3	8.2	65210	26.14	75.09	− 0.538	53	55
12	*B*. *acidocaldarius*	Q43880	546	59769.6	5.47	102110	29.95	73.97	− 0.372	59	48
13	*Bacillus sp*. *EA1*	Q59223	546	59812.7	5.39	102110	29.08	74.51	− 0.363	60	48
14	*Clostridium putrefaciens*	A0A381J9B8	553	61718.7	4.9	77255	31.24	77.72	− 0.444	73	49
15	*Enterococcus faecalis*	Q833V7	510	55503.8	4.97	48250	22.68	81.98	− 0.348	67	43
16	*Erwinia carotovora*	Q99132	347	38828.4	5.48	51005	38.81	79.31	− 0.422	42	25
17	*Legionella longbeachae*	P55110	529	58712.6	5.71	93880	36.35	65.46	− 0.435	59	46
18	*Legionella pneumophila*	P21347	543	60704.1	5.27	95370	34.62	64.83	− 0.491	66	47
19	*Listeria monocytogenes*	P23224	510	57411	6.61	62020	27.85	80.53	− 0.477	68	65
20	*Pseudomonas aeruginosa*	P14756	498	53687.1	6.28	65000	26.9	73.49	− 0.271	50	46
21	*Renibacterium salmoninarum*	P55111	548	56378.6	5.2	47245	17.53	79.73	− 0.095	47	40
22	*Staphylococcus aureus*	P81177	509	56320.9	5.02	56730	23.75	68.21	− 0.732	80	56
23	*Staphylococcus epidermidis*	P0C0Q3	507	55813.5	5.35	55240	29.37	69.45	− 0.691	68	56
24	*Streptomyces lividans*	D6EEH2	547	56812.3	4.75	84145	19.32	75.43	− 0.235	71	43
25	*Serratia marcescens*	Q06517	352	38515.3	10	49640	52.68	65.26	− 0.698	29	48
26	*Vibrio proteolyticus*	Q00971	609	66362.5	5.07	88045	30.55	64.84	− 0.359	64	45
27	*Vibrio anguillarum*	P43147	611	66726.5	5.7	92055	30.83	68.43	− 0.292	53	44
28	*Vibrio aestuarianus*	A8JNY9	611	66344.9	5.33	93545	30.57	72.9	− 0.252	54	41
29	*Vibrio vulnificus*	O06694	606	65613.9	6.17	93085	32.45	66.67	− 0.341	54	48
30	*Vibrio cholerae*	P24153	609	65891.2	5.26	92055	25.18	68.82	− 0.25	53	39
31	*Vibrio splendidus*	Q76LC2	607	65879	5.74	88045	30.39	68.29	− 0.332	56	46

### Primary sequence analysis

**Fig. 3:**
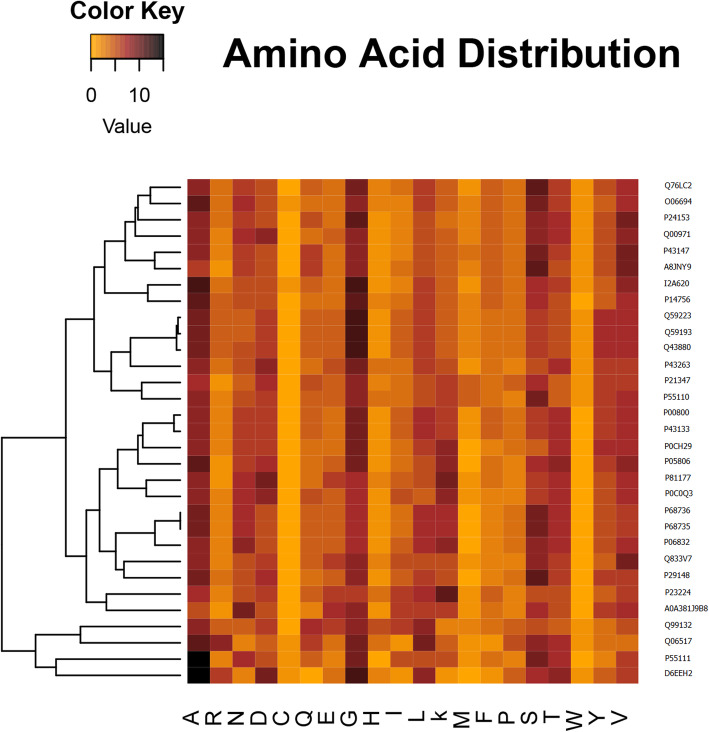
Amino acids distribution in 31 different bacterial M4 metalloprotease enzymes

The primary structure analysis of M4 metalloprotease enzymes included amino acid distribution, motif, and domain analysis. The amino acid distribution was represented as a heatmap in Fig. 3 which showed that the most abundant amino acid was Ala and the least common amino acid was cysteine.

**Fig. 4:**
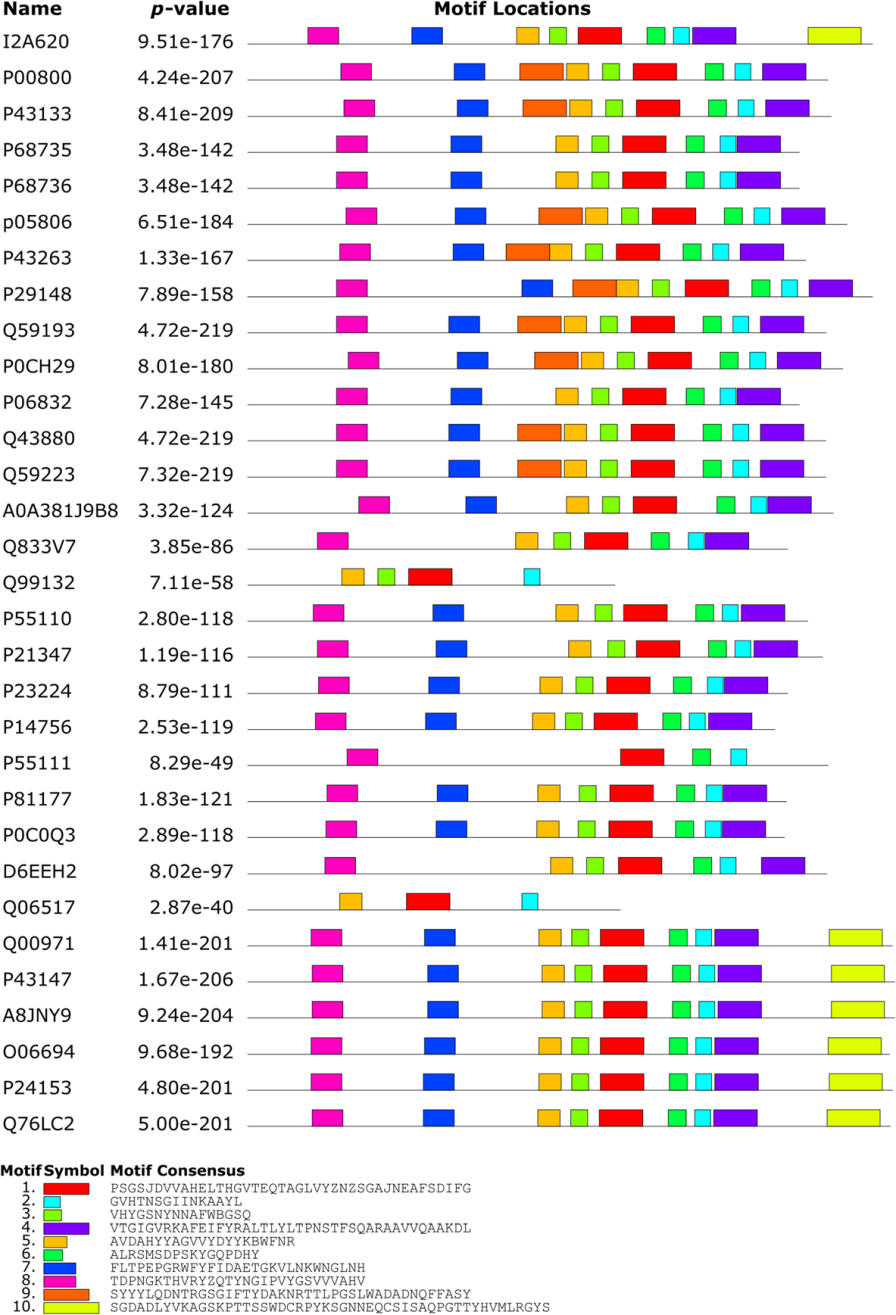
Occurrence of ten tandem motifs among 31 different bacterial M4 metalloprotease proteins subjected to MEME

A total of 10 motifs were observed in 31 sequences when subjected to MEME and depicted in Fig. 4. The motifs with width and best possible match amino acid sequences were given in Table 2. A set of 41 amino acid residues, i.e., PSGSJDVVAHELTHGVTEQTAGLVYZNZSGAJNEAFSDIFG representing motif 1 was uniformly observed in all sequences revealing its identity with the peptidase_M4 and Peptidase_M4_C domains (Table 2). The order of amino acid residues “HELTH” in this sequence was associated with the active site of the enzyme. Motif 8 was uniformly observed in most of the proteases sequences except Q99132 (*Erwinia carotovora*) and Q06517 (*Serratia marcescens*) represented a signal peptide FTP domain which prevented premature activation of proteases [51]. The region of motif 7 was likely to have a protease inhibitory function since it belonged to the pepSY domain [52]. In case of motifs 3, 5 and 9 belonging to Peptidase_M4 domain were associated with the catalysis of the enzyme. The motifs 2, 4, and 6, belonging to Peptidase_M4_C domain, were important in order to the presence of alpha helix related with the flexibility of protein conformation and protein function. Motif 10 was observed only in five species from genus *vibrio* (Q00971, *V*. *proteolyticus*; P43147, *V*. *anguillarum*; A8JNY9, *V*. *aestuarianus*; O06694, *V*. *vulnificus*; and P24153, *V*. *cholerae*) along with I2A62, *Aeromonas hydrophila*.

**Table 2:** Distribution of different motifs with best possible match amino acid sequences along with functional domains

Motif no.	Width	Best possible amino acids	Domain
1	41	PSGSJDVVAHELTHGVTEQTAGLVYZNZSGAJNEAFSDIFG	Peptidase_M4 and Peptidase_M4_C
2	15	GVHTNSGIINKAAYL	Peptidase_M4_C
3	16	VHYGSNYNNAFWBGSQ	Peptidase_M4
4	41	VTGIGVRKAFEIFYRALTLYLTPNSTFSQARAAVVQAAKDL	Peptidase_M4_C
5	21	AVDAHYYAGVVYDYYKBWFNR	Peptidase_M4
6	17	ALRSMSDPSKYGQPDHY	Peptidase_M4_C
7	29	FLTPEPGRWFYFIDAETGKVLNKWNGLNH	PepSY
8	29	TDPNGKTHVRYZQTYNGIPVYGSVVVAHV	FTP
9	41	SYYYLQDNTRGSGIFTYDAKNRTTLPGSLWADADNQFFASY	Peptidase_M4
10	50	SGDADLYVKAGSKPTTSSWDCRPYKSGNNEQCSISAQPGTTYHVMLRGYS	PPC

### Secondary structure analysis

The predicted secondary structure composition of M4 metalloprotease was determined using the NPS@ server which generated a consensus report from twelve secondary structure prediction methods. The secondary structure prediction server revealed that the enzyme is dominated by 41.64% of amino acid in random coils along with 32.12% of amino acids resided in α-helices, while 20.36% of residues were in extended sheet. Finally, less amount of the amino acids was found in extended sheet region of 5.88% (Table 3).

**Table 3:** Predicted consensus secondary structure content and predicted disulfide patterns of M4 metalloproteases

S.N.	Bacteria name	UniProtKB	Alpha helix	Extended strand	Beta turn	Random coil	Disulfide bridges prediction
1	*Aeromonas hydrophila*	I2A620	28.98	23.39	7.12	40.51	214–550, 240–452, 481–561
2	*Bacillus thermoproteolyticus*	P00800	35.58	20.99	4.74	38.69	None
3	*B*. *stearothermophilus*	P43133	32.67	20.33	5.26	41.74	None
4	*B*. *subtilis subsp*. *Amylosacchariticus*	P68735	27.64	20.54	6.14	45.68	None
5	*B*. *subtilis*	P68736	27.64	20.54	6.14	45.68	None
6	*B*. *cereus*	P05806	30.92	20.32	4.95	43.82	None
7	*B*. *brevis*	P43263	33.02	20.87	5.69	40.42	None
8	*B*. *polymyxa*	P29148	30.68	20.68	4.58	44.07	None
9	*B*. *caldolyticus*	Q59193	32.6	20.51	4.76	42.12	None
10	*B*. *megaterium*	P0CH29	31.14	19.75	5.16	43.95	None
11	*B*. *amyloliquefaciens*	P06832	32.44	19	4.8	43.76	None
12	*B*. *acidocaldarius*	Q43880	31.14	21.25	5.13	42.49	None
13	*Bacillus sp*. (*strain EA1*)	Q59223	32.42	22.53	5.13	39.93	None
14	*Clostridium putrefaciens*	A0A381J9B8	33.45	19.89	5.79	40.87	None
15	*Enterococcus faecalis*	Q833V7	30.39	23.53	5.88	40.2	None
16	*Erwinia_carotovora*	Q99132	44.09	12.97	6.05	36.89	None
17	*Legionella longbeachae*	P55110	35.92	18.53	7.37	38.19	243–270, 349–498
18	*Legionella pneumophila*	P21347	32.04	19.15	7.55	41.25	None
19	*Listeria monocytogenes*	P23224	30.39	21.96	5.1	42.55	None
20	*Pseudomonas aeruginosa*	P14756	37.15	18.67	7.83	36.35	227–255, 467–494
21	*Renibacterium salmoninarum*	P55111	35.58	15.33	6.75	42.34	258–336, 519–535
22	*Staphylococcus aureus*	P81177	31.43	20.43	5.3	42.83	None
23	*Staphylococcus epidermidis*	P0C0Q3	33.73	20.71	4.93	40.63	None
24	*Streptomyces lividans TK24*	D6EEH2	27.24	21.57	5.48	45.7	336–438, 338–481
25	*Serratia marcescens*	Q06517	37.78	12.22	5.68	44.32	139–295
26	*Vibrio proteolyticus*	Q00971	30.21	22.66	6.73	40.39	231–570, 257–581, 473–502
27	*Vibrio anguillarum*	P43147	30.61	21.93	5.73	41.73	260–572, 476–583
28	*Vibrio aestuarianus*	A8JNY9	29.3	22.59	7.53	40.59	234–260, 476–572, 505–583
29	*Vibrio vulnificus*	O06694	30.03	21.45	6.27	42.24	231–257, 473–502, 569–580
30	*Vibrio cholerae O1*	P24153	28.74	23.97	7.06	40.23	231–473, 257–581
31	*Vibrio splendidus*	Q76LC2	30.81	23.06	5.6	40.53	230–568, 256–579

**Fig. 5:**
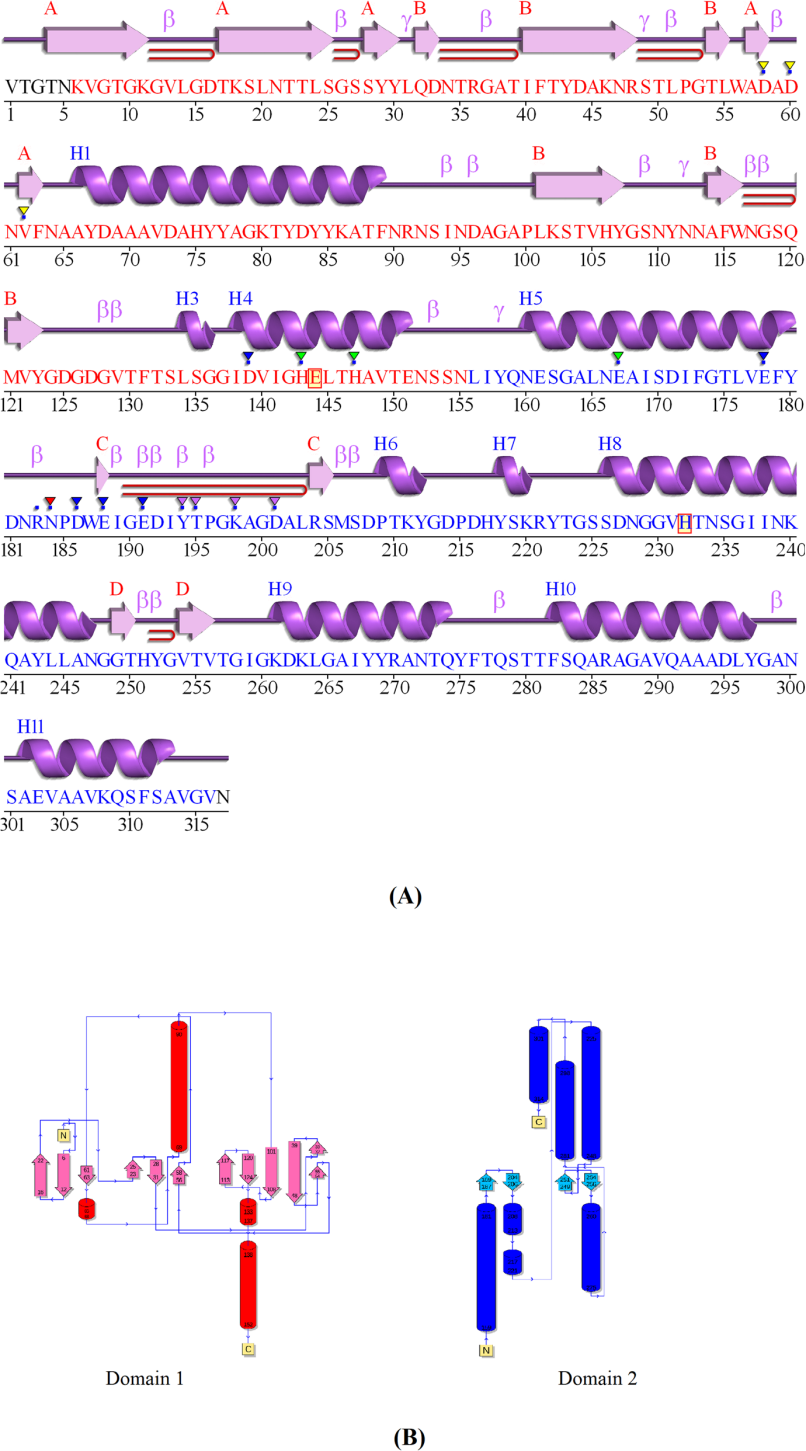
Schematic and topology diagrams showing the secondary structural elements in the M4 metalloprotease protein (PDB ID 1NPC), was calculated using the PDBsum tool. **a** α-Helices were labeled with the letter “H”, and β-strands were lettered in uppercase. β, γ, and hairpin turns were also labeled. **b** Helices were represented as cylinders and β-strands as arrows

A more detailed analysis of the secondary structural elements was performed using the PDBsum tool. Here, the amino acid sequence of M4 metalloprotease from *B*. *cereus* was taken as template which is also known as Bacillolysin. The predicted secondary structures generated by the PDBsum tool in Fig. 5a were generally in an idea of the “structural coverage”, how much the protein sequence of *B*. *cereus* M4 metalloprotease was actually represented by the 3D structure [53]. The secondary structure displaying 11 helices (H1–H11) involved in 15 helix-helix interactions, while 4 β-sheet motifs composed of 15 β-strands. According to the diagram, the catalytic site lid ^143^HELTH^147^ was located within helix H4. This was also observed in the 3D structure. The topology of *B*. *cereus* M4 metalloprotease was illustrated in Fig. 5b which showed the arrangement and connectivity of the helices and strands in protein. Where the protein chain consisted of two domains, a C-terminal domain and an N-terminal domain were both folded into a mixed α/β topology.

Disulfide bonds play an important role in folding and stabilizing the unfolded form of the protein by lowering the entropy. Possible disulfide linkages in the primary structure were determined using CYS_REC was represented in Table 3. In most of the cases disulfide bridges were absent, as the prevalence of cysteine residues were very poor (Fig. 3).

### Tertiary structure analysis

**Fig. 6:**
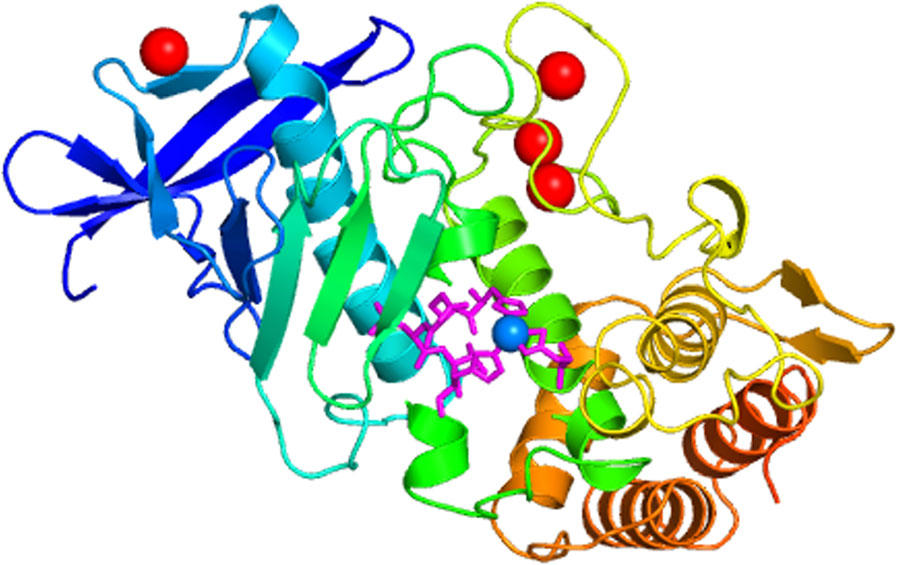
Predicted 3D structure of *B*. *cereus* M4 metalloprotease protein. The model was generated with SWISS-MODEL using PDB template 1NPC. PyMOL was used to visualize the model. The active side lid was shown as a pink loop, Ca^2+^ binding and Zn^2+^ binding site was illustrated as red and blue dots, respectively

**Fig. 7:**
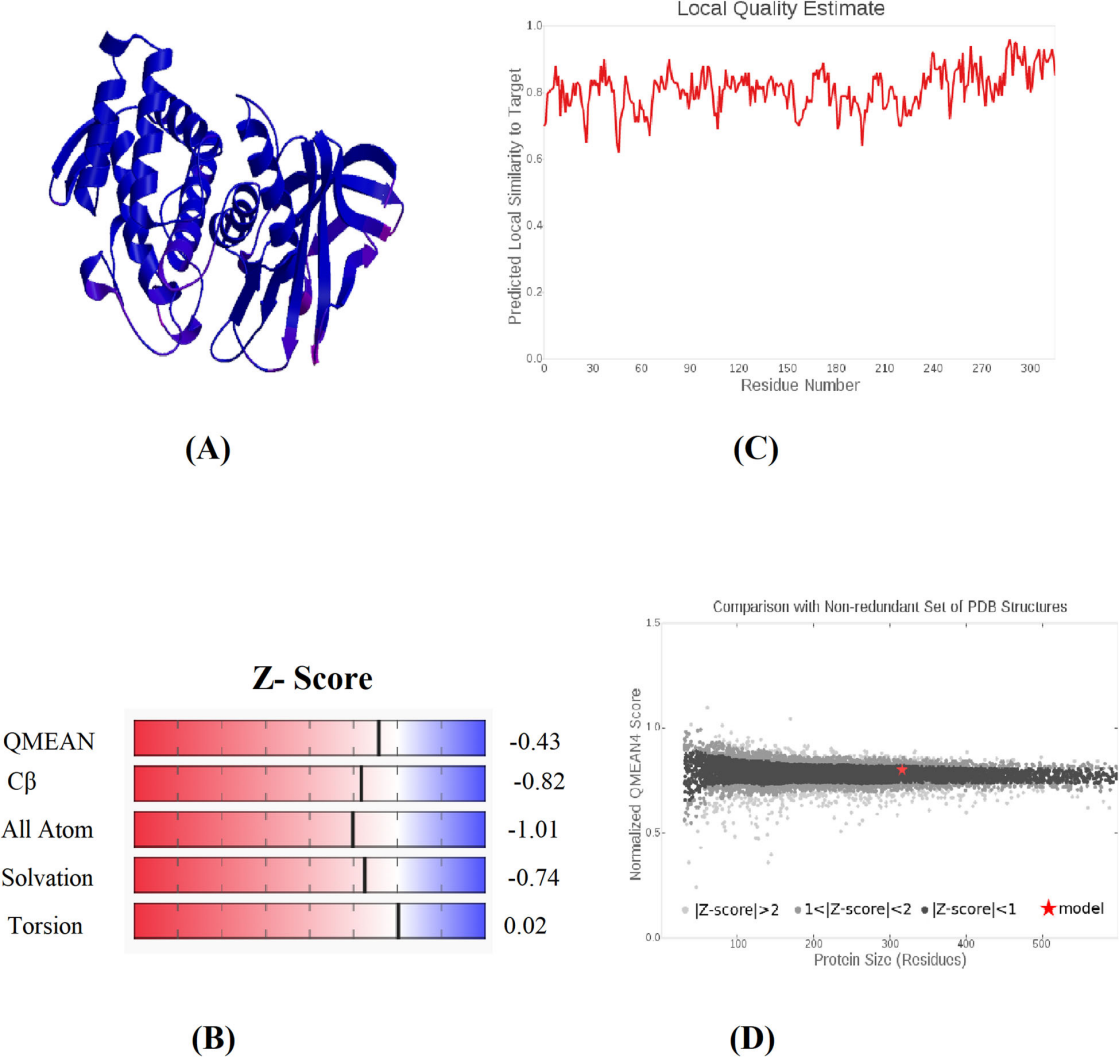
Quality analysis of predicted model from QMEAN4 server. **a** QMEAN PDB 3D model of M4 metalloprotease protein (PDB ID 1NPC) structure. **b** The *z*-scores of the QMEAN terms of the protein model PDB ID 1NPC. **c** Graphical presentation of estimation of local quality. **d** Graphical presentation of estimation of absolute quality of model (PDB ID 1NPC)

**Fig. 8:**
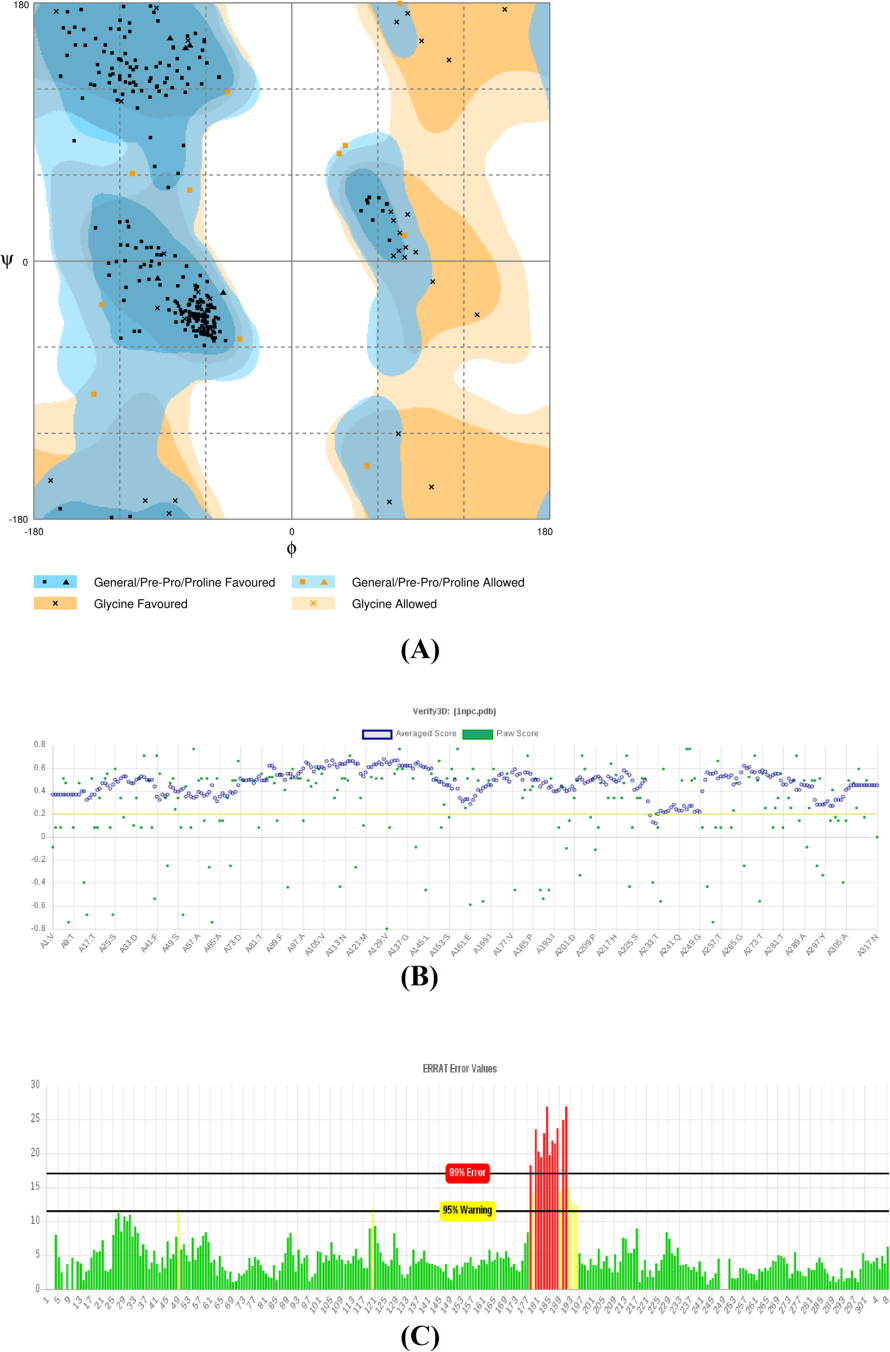
Evaluation of bacterial M4 metalloprotease protein (PDB ID 1NPC) form SAVES server. **a** Ramachandran plot. **b** Verify 3D graph. **c** ERRAT generated results

The tertiary structure of M4 metalloprotease was generated with the SWISS-MODEL using *B*. *cereus* M4 metalloprotease (PDB ID: 1NPC) as a template and PyMOL was used to visualize the model (Fig. 6). The active side lid was shown as a pink loop, four Ca^2+^ binding site was illustrated as pink and a Zn^2+^ binding site was as blue. This model was further verified by QMEAN4 and SAVES server. QMEAN PDB result was represented in Fig. 7 and depicted the proper folding of protein into a compact three-dimensional field. Ramachandran plot measured the accuracy of protein model and the results were narrated in Fig. 8a. The profile score above zero in the Verify3D graph correspond to the acceptable environment of the model, in Fig. 8b. ERRAT-verified protein structure and the result depicted in Fig. 8c.

### Functional analysis

**Fig. 9:**
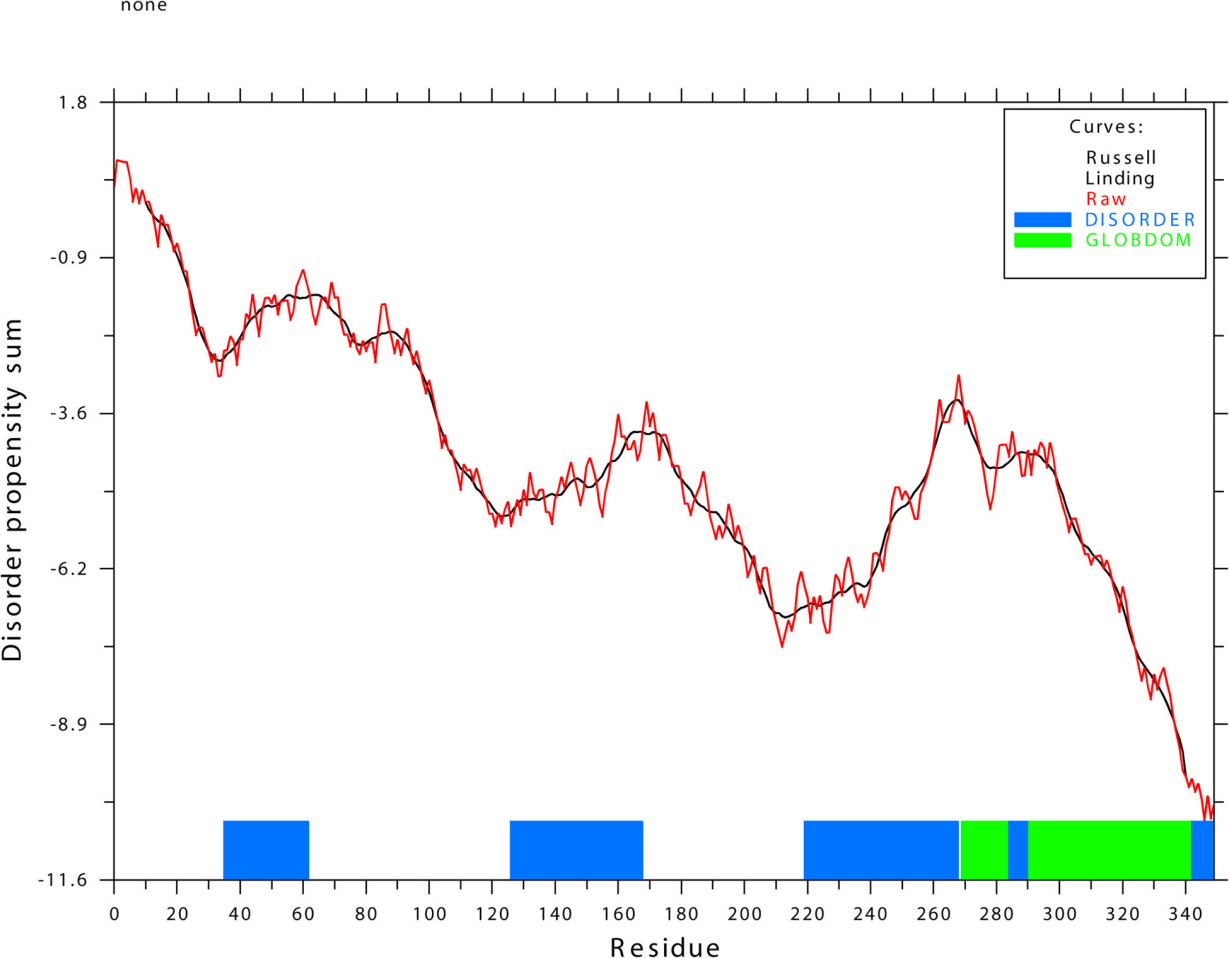
Globplot of M4 metalloprotease protein (PDB ID 1NPC). Five regions appeared to be in a disordered state, marked as blue-colored sections, and green-colored regions were globular or ordered domains.

For functional analysis, the query sequence taken was the amino acid sequence of M4 metalloprotease from *B*. *cereus* (PDB ID: 1NPC). Here, transmembrane helix prediction analyzed by TMHMM server 2.0 showed that no transmembrane helix presents in the protein. 5 disorder regions were identified by GlobPlot and the regions were from amino acid number 1–29, 93–135, 186–235, 251–257, and 309–317. In Fig. 9, the blue-colored sections were disordered regions and green-colored regions were globular or ordered domains. Protease digestion is a useful process that is used to know proper metabolism, enzymatic digestion and simplification of high order protein structure. According to results from peptide cutter, there were several cleavage sites for 21 different digestive enzymes for the amino acid sequence of M4 metalloprotease from *B*. *cereus*. Table 4 summarized the results obtained by the peptide cutter tool which indicated that total numbers of cleavages were found to be 633.

**Table 4:** Cleavage of amino acid residues by different enzymes generated by ExPASy peptide cutter

Name of enzyme	No of cleavages	Positions of cleavage sites
Arg-C proteinase	8	36, 48, 91, 183, 204, 221, 270, 286
Asp-N endopeptidase	23	15, 32, 43, 57, 59, 67, 72, 82, 95, 124, 126, 138, 170, 180, 185, 191, 200, 207, 213, 215, 226, 261, 294
Asp-N endopeptidase + N-terminal Glu	31	15, 32, 43, 57, 59, 67, 72, 82, 95, 124, 126, 138, 143, 150, 160, 166, 170, 177, 180, 185, 187, 190, 191, 200, 207, 213, 215, 226, 261, 294, and 302
BNPS-Skatole	3	56, 116, and 187
CNBr	2	121 and 206
Caspase1	1	58
Chymotrypsin-high specificity	37	29, 30, 41, 43, 56, 63, 67, 76, 77, 82, 84, 85, 89, 107, 111, 115, 116, 123, 131, 158, 173, 179, 180, 187, 194, 212, 218, 222, 243, 252, 268, 269, 275, 276, 282, 297, and 311
Chymotrypsin-low specificity	62	14, 20, 24, 29, 30, 31, 41, 43, 55, 56, 63, 67, 75, 76, 77, 82, 84, 85, 89, 101, 106, 107, 111, 115, 116, 121, 123, 131, 134, 143, 145, 147, 156, 158, 165, 173, 176, 179, 180, 187, 194, 203, 206, 212, 217, 218, 222, 232, 243, 244, 245, 251, 252, 264, 268, 269, 275, 276, 282, 296, 297, and 311
Clostripain	8	36, 48, 91, 183, 204, 221, 270, and 286
Formic acid	23	16, 33, 44, 58, 60, 68, 73, 83, 96, 125, 127, 139, 171, 181, 186, 192, 201, 208, 214, 216, 227, 262, and 295
Glutamyl endopeptidase	8	144, 151, 161, 167, 178, 188, 191, and 303
Hydroxylamine	3	117, 228, and 247
Iodosobenzoic acid	3	56, 116, and 187
LysC	14	6, 11, 18, 46, 80, 86, 102, 198, 211, 220, 240, 261, 263, and 308
LysN	14	5, 10, 17, 45, 79, 85, 101, 197, 210, 219, 239, 260, 262, and 307
Pepsin (pH 1.3)	45	14, 19, 23, 24, 30, 31, 40, 41, 51, 54, 55, 62, 63, 89, 100, 114, 115, 130, 131, 133, 134, 144, 155, 156, 164, 165, 172, 173, 175, 176, 178, 179, 202, 203, 243, 244, 245, 264, 275, 276, 281, 282, 295, 296, and 311
Pepsin (pH > 2)	81	14, 19, 23, 24, 28, 29, 30, 31, 40, 41, 42, 43, 51, 54, 55, 56, 62, 63, 66, 67, 75, 76, 81, 83, 84, 85, 89, 100, 106, 107, 110, 111, 114, 115, 116, 122, 123, 130, 131, 133, 134, 144, 155, 156, 157, 158, 164, 165, 172, 173, 175, 176, 178, 179, 180, 187, 193, 202, 203, 211, 212, 217, 218, 243, 244, 245, 251, 252, 264, 267, 268, 269, 274, 275, 276, 281, 282, 295, 296, 297, and 311
Proteinase K	159	1, 2, 4, 7, 9, 13, 14, 17, 20, 22, 23, 24, 29, 30, 31, 35, 38, 39, 40, 41, 42, 43, 45, 50, 51, 54, 55, 56, 57, 59, 62, 63, 65, 66, 67, 69, 70, 71, 72, 74, 76, 77, 78, 81, 82, 84, 85, 87, 88, 89, 94, 97, 99, 101, 104, 105, 107, 111, 114, 115, 116, 122, 123, 129, 130, 131, 132, 134, 138, 140, 141, 144, 145, 146, 148, 149, 150, 151, 156, 157, 158, 161, 164, 165, 167, 168, 169, 172, 173, 175, 176, 177, 178, 179, 180, 187, 188, 189, 191, 193, 194, 195, 199, 202, 203, 210, 212, 218, 222, 223, 231, 233, 237, 238, 242, 243, 244, 245, 246, 250, 252, 254, 255, 256, 257, 259, 264, 266, 267, 268, 269, 271, 273, 275, 276, 277, 280, 281, 282, 285, 287, 289, 290, 292, 293, 294, 296, 297, 299, 302, 303, 304, 305, 306, 307, 311, 313, 314, and 316
Staphylococcal peptidase I	8	144, 151, 161, 167, 178, 188, 191, and 303
Thermolysin	78	6, 12, 13, 19, 23, 30, 37, 39, 40, 54, 56, 61, 62, 64, 65, 69, 70, 71, 77, 86, 88, 93, 100, 104, 113, 114, 120, 121, 128, 130, 133, 137, 140, 147, 148, 155, 156, 163, 164, 168, 172, 175, 176, 198, 202, 205, 230, 236, 237, 241, 243, 244, 245, 253, 255, 258, 263, 265, 266, 270, 275, 281, 284, 286, 288, 289, 291, 292, 293, 298, 301, 304, 305, 306, 310, 312, 313, and 315
Trypsin	22	6, 11, 18, 36, 46, 48, 80, 86, 91, 102, 183, 198, 204, 211, 220, 221, 240, 261, 263, 270, 286, and 308

**Fig. 10:**
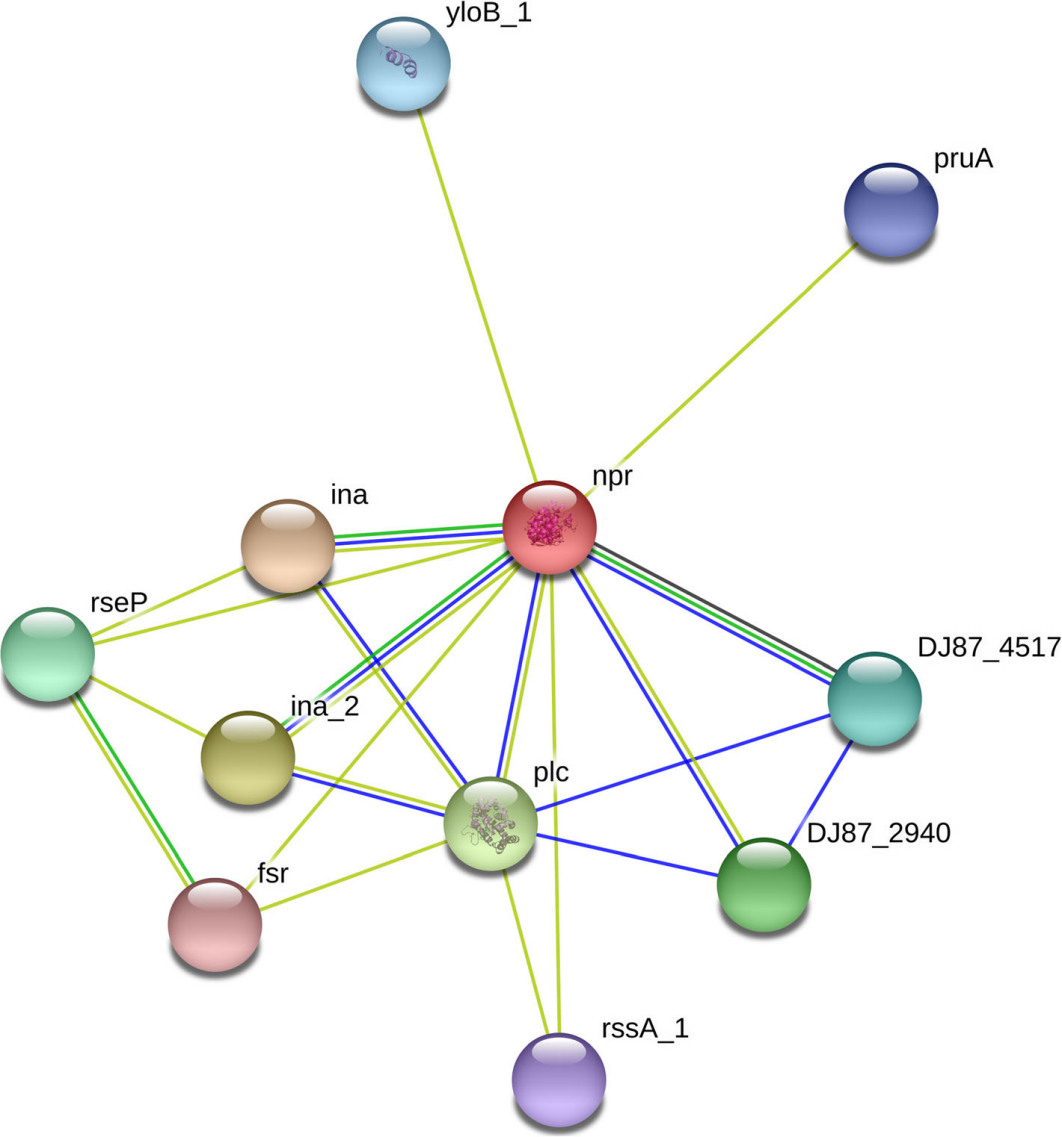
Protein-protein interaction network of M4 metalloprotease (PDB ID 1NPC) detected through STRING (PDB ID 1NPC). The red node (npr protein) represented M4 metalloprotease (PDB ID 1NPC) and other nodes represented its predicted functional partners. The minimum interaction score was set to medium confidence (0.400)

The protein-protein interacting partners of bacillolysin from *B*. *cereus* was generated through STRING 11.0 and presented in Fig. 10. STRING forecasted confidence scores (0.771–0.591) which indicated the functional network among the set of proteins of a given organism. M4 metalloprotease of *B*. *cereus* (npr) was predicted to be interacting with 10 proteins, namely ina, ina_2, plc, DJ87_2940, rseP, DJ87_4517, yloB_1, pruA, rssA_1, and fsr in different manner. The STRING database analysis depicted that npr p*rotein-protein interaction* (PPI) network comprised of 11 nodes connected with 20 different edges. Whereas, expected number of edges was observed to be 11; while the average node degree score was found to be 3.64 i.e., one node had at least 3.64 interacting nodes. Average local clustering coefficient was predicted to be 0.732 and PPI enrichment *p*-value was observed as 0.00703.

### Prediction of active sites

**Fig. 11:**
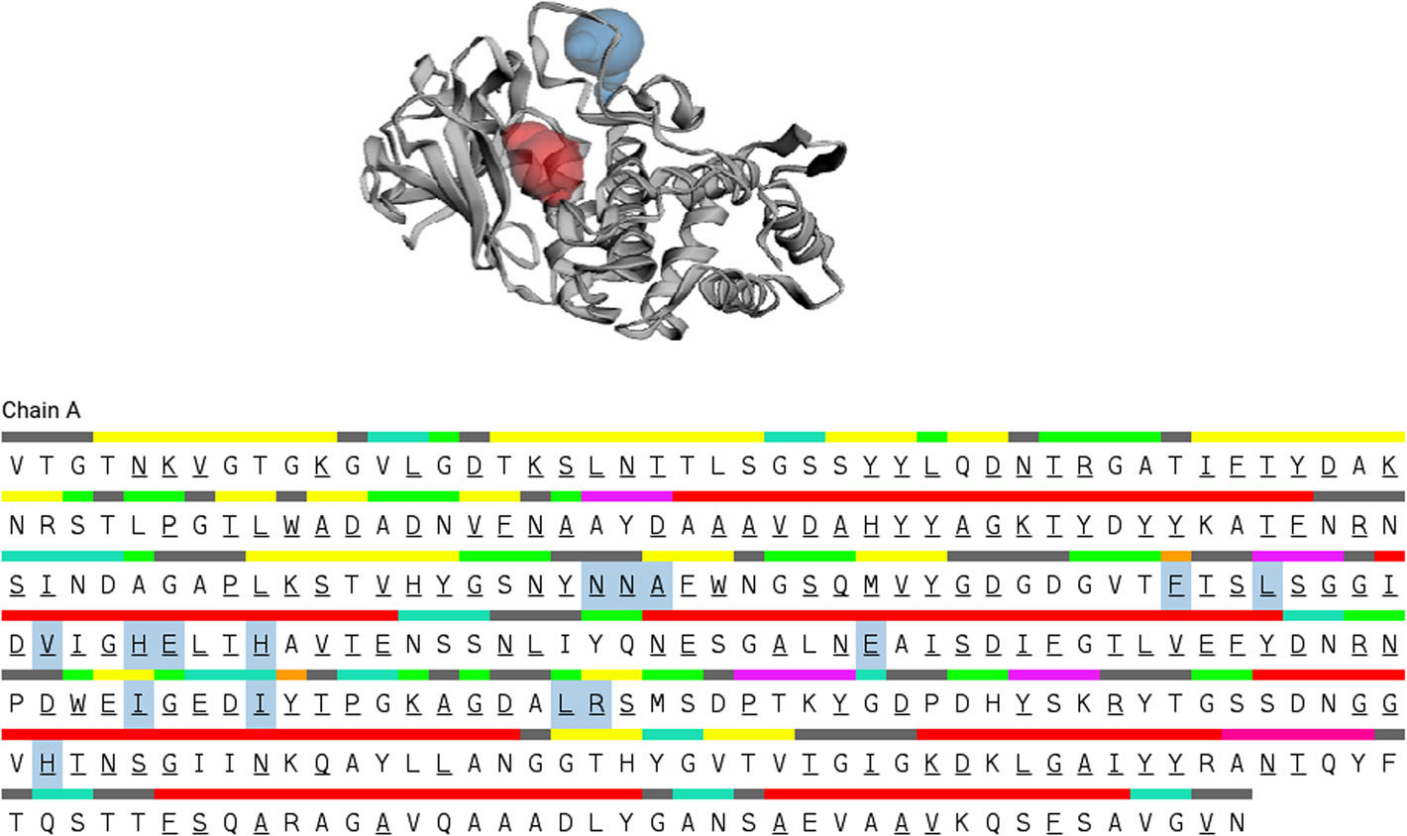
Active site information of M4 metalloprotease (PDB ID 1NPC) obtained from CASTp serve. **a** Visualization of pocket automatically identified on the structure of the active site of the protein. **b** Active site information. Green color illustrated the amino acids position in active site

CASTp 3.0 server was used to identify the possible active site for ligand in the M4 metalloprotease of *Bacillus cereus* (PDB 1NPC). In the present study, the surpass active site area of the enzyme in addition to the number of amino acids occupied in it were also reported. The preeminent active site was found the largest pocket with 103.076 areas and a volume of 76.471 amino acids (Fig. 11). Many functionally important residues were located in this pocket, including three residues His 143, His 147, and Glu 167 in zinc-binding site and two residues, Glu 144 and His 232, in the catalytic site which were essential for the most common HEXXH zinc-binding motif in metalloprotese. Another pocket with an area of 49.147 and a volume of 32.345 amino acids comprising three residues, Glu 167, Asp 171, and Glu 191, in Calcium 2 binding site; four residues, Glu 178, Asn 184, Asp 186, and Glu 191, in Calcium 3 binding site; and four residues, Tyr 194, Thr 195, Lys 198, and Asp 201 in Calcium 4 binding site, since they have also been reported to be essential for the functioning of other bacterial organisms [2, 54, 55]. The 3D representation of pockets was shown in Fig. 11 with largest pocket in red color and other pocket in blue color.

### Discussion

The aim of the study was to identify the structure and properties of M4 metalloprotease proteins using bioinformatics tools. The present study primarily determined the global similarities among the compared proteins. In amino acid sequence alignment of 31 M4 metalloproteases, a conserved region (HELTE) was observed (Fig. 1). The presence of two His and one Glu is important for activity in all the metallopeptidases that carry the HEXXH zinc-binding motif. In case of metallopeptidases having two catalytic metal ions, Ca^2+^ and Zn^2+^, along with two residues, a glutamate and an aspartate are also essential [56]. The existence of conserved amino acid plays significant role in confirmation of protein and helix coil transition. Gly and Pro frequently coincide with the extremities of well-structured beta strands or alpha helices. His and Ser are often involved in catalytic sites, especially in proteases. Charged amino acids like Asp, Glu, and Arg are mostly involved in ligand binding. Highly conserved columns might indicate a salt bridge inside the core of the protein [57].

In this study, phylogenetic trees were constructed using both amino acid sequence versus gene sequences to find if there was any correlation among the taxa in terms of their protein sequences compared with respective cDNA. Results obtained from the evolutionary tree (Fig. 2) implied that metalloproteases from different bacterial species appeared to be related to each other and clustering in distinct groups based on its source organisms and nature of the mechanism of enzymatic activity. Thus, it can be inferred that the bacterial strains might be diverged from a common evolutionary ancestor.

A physicochemical analysis of the protein sequence was determined by the Expasy server’s ProtParam tool. It revealed all the proteins have negative GRAVY scores which attested to their solubility in hydrophilic solvents and substantiated by earlier studies [58–60]. Average extinction coefficient 78371.13 referred the quantity of light that may be absorbed by protein in 280 nm. In theory, when the pI value of a protein exceeds 7, it is characterized as alkaline in nature and the value of below 7 indicates the acidic nature. In this study, the pI values of all the proteins showed broad range of 4.88–10 indicating diverse nature of protein. Metalloproteases from all the selected bacteria except *P*. *aeruginosa* were found to be stable with instability index less than 40, which justified by the previous studies [58, 59]. The aliphatic index of a protein is used to measure the relative volume of protein occupied by amino acids in aliphatic side chain [61] and higher value of aliphatic index is considered a positive factor of increased thermo stability. Here, all strains showed high aliphatic index (Ai) of 64.83–81.98 which indicated the thermostability of the proteins [62].

Based on the amino acid distribution, the most abundant amino acid was Ala which accounted for 9.3% of the enzyme’s primary structure (Fig. 3). The least common amino acid was cysteine. Other predominant amino acids were found to be Gly (9.1%), Ser (7.8%), Val (7.5%), Asp (6.6%), Lys (6.6%), and Thr (6.5%). Ala is very rare to be dug inside the protein core due to its hydrophobic nature so that it has less tendency to contact with water. On the other hand, due to not having a side chain, Gly mostly occupied the surface of the protein providing high flexibility to the polypeptide chain. The presence of significant amount of hydrophilic amino acids such as Ser and Thr represented the protein as extracellular in nature. As Asp is charged and polar amino acid, it might be occurred on the surface of proteins and involved in salt bridge. Being positively charged, Lys preferred to be in the side chain of proteins and formed salt bridge [63]. The domain analysis exposed different conserved site present in M4 metalloprotease from bacterial sources. The presence of common and unique domains among different proteases might confer their structural flexibility, which directly influences functional activity of proteases. These conserved regions might be utilized for designing primers for PCR-based amplification and cloning of these proteases genes from different bacterial species.

In this study, *B*. *cereus* M4 metalloprotease (1NPC) was selected as a representative species for describing secondary and tertiary structures of the M4 metalloprotease proteins. Family M4 contains a wide range of extracellular thermolysin. Among them, the 3D structures are known for thermolysin from *Bacillus cereus* (1NPC) [64] and *B*. *thermoproteolyticus* (1KEI) [65]. Thermolysin from *B*. *thermoproteolyticus* have been well-characterized structurally and enzymatically, i.e., its primary and tertiary structures and substrate-binding site. But very little information is available for thermolysin from *Bacillus cereus*. This was the reason behind the selection of *B*. *cereus* M4 metalloprotease (PDB ID: 1NPC). Results generated by secondary structure prediction tool SOPMA showed the abundance of coiled region (41.64%) indicated higher conservation and stability of the model 1NPC [66, 67]. Information from PDBsum aided in determining the overall structural organization of proteins and predicting protein pockets for ligand binding. Thus, the secondary structure arrangement of the protein could help in the prediction of tertiary structures. On the other hand, secondary structural elements prediction may overcome the limitations of X-ray crystallography and NMR for tertiary structure of protein. Crystallization of few proteins is very difficult task by X-ray crystallography and NMR is restricted to relatively small protein molecules. Moreover, Roy et al. [68] reported that prediction of secondary structural elements was vital for detection conformational changes within the protein of interest.

The protein 3D model gained from SWISS-MODEL workspace was evaluated by both QMEAN4 and SAVES server. QMEAN output estimated geometrical aspects of the protein structure that characterized the global arrangement of variable residues of protease. According to Benkert et al., the QMEAN *z*-score determines of the absolute quality of a model by relating it to the reference structures solved by X-ray crystallography [69]. In Fig. 7c, the *z*-scores of the QMEAN terms of the protein model were − 0.82, − 1.01, − 0.74, and 0.02 for Cβ interaction energy, all atom energy, salvation energy, and torsion angle energy, respectively. These scores implied that the predicted protein model could be considered a quality model. Furthermore, for the estimation of perfect quality of the model, the QMEAN server relates the query model with a representative set of high-resolution X-ray structures of similar size and the resulting QMEAN *z*-score is an extent of degree of nativeness of the particular structure [70]. For high-resolution models, the average *z*-score is ‘0’. Here, QMEAN *z*-score for the query model was − 0.43, which was lower than the standard deviation ‘1’ from the mean value ‘0’ of good models, so this result indicated that the estimated model was comparable to the high-resolution models. Again, from the estimation of absolute quality of modeled protein in Fig. 7d, the dark zone indicated that the model had a score < 1. Normally, models considered good are expected to position in the dark zone. In this case, the model was considered to be good according to their position in the dark zone which was showed as red marker. This finding was similar with Hasan et al. [71]. The structure of *B*. *cereus* M4 metalloprotease was further verified using SAVES server. Ramachandran plot, verify 3D, and ERRAT were evaluated from SAVES. These methods were essential for understanding 3D protein models and the estimation of their accuracy. According to the Ramachandran plot generated with the RAMPAGE server, 96.5% of residues are found in the favored region, while 3.5% of amino acids reside in the allowed region (Fig. 8). According to Yadav et al., > 90% of the residues residing in favored region implied the characteristics of a good quality model [72]. Thus, a good stereo-chemical quality of the model was ensured by the Ramachandran plot, whereas the 3D model passed the Verify 3D with 99.05% as 99.05% of its residues had an average 3D-1D score ≥ 0.2. According to Verify 3D server, at least 80% of the amino acids has scored ≥ 0.2 in the 3D/1D profile would be acceptable. Again in ERRAT, the structure verification algorithm interpreted the overall quality of the model with the resulting score 93.33; this score denoted the percentage of the protein that fell below the rejection limit of 95% [73]. So ERRAT program also verified the protein 3D structure as acceptable. From the above analyses, it was confirmed that predicted structure of the protein was good, stable, reliable, and consistent.

TMHMM tool indicates there was no transmembrane domain present in the protein, confirming the extracellular production nature of *B*. *cereus* M4 metalloprotease. Similar type of observation regarding the TMHMM result was also shown by Dutta et al. [63]. GlobPlot tool was used to identify disorder regions. Disorder of protein is denoted as a high degree of flexibility in polypeptide chain and lack of regular secondary structure [74]. In Fig. 9, the blue-colored sections were disordered regions. Many proteins are intrinsically found disordered in vivo. Disordered regions are important because many intrinsically disordered proteins exist as unstructured and become structured when bound to another molecule [75, 76]. According to the result from peptide cutter tool, a total of 633 cleavages were obtained which might be helpful to carry out experiments with a portion of a protein, to separate the domains in a protein, and to remove a tag protein when expressing a fusion protein.

Protein-protein interaction (PPI) networks are used to identify the complex molecular mechanisms and pathways to gain basic knowledge of diseases. PPI network demonstrated that bacillolysin interacted with ten other proteins in a high confidence score, among them the closest annotated interacting protein having the shortest node with score of 0.77 was found ina. Then, ina_2, immune inhibitor A, was found having the score of 0.768 that functioned in degrading host tissue proteins with broad substrate specificity [77]. Again, plc (score 0.662), which stood for Phospholipase C, was involved in hemolysis and cell rupture [24]. DJ87_2940 (score 0.647) was an enterotoxin; a non-hemolytic protein, yolB_1 (score 0.595), was a calcium-translocating P-type ATPase which functioned to transport a variety of different compounds, including ions and phospholipids across a membrane. PruA (score 0.591) was a putative delta-1-pyrroline-5-carboxylate dehydrogenase which was involved in antibiotic biosynthesis pathway. From PPI network analysis, it can be estimated that bacillolysin from *B*. *cereus* may be a part of its immune system.

Analysis of protein structures for active site often considers as the starting point in the protein-ligand docking studies. The active site of an enzyme comprises a substrate-binding site and a catalytic site. The enzyme binds with a specific substrate in order to catalyze a chemical reaction, whereas the catalytic site occurs next to the binding site, carrying out the catalysis. Some enzymes require the help of cofactors for their activities. Mostly cofactors are connected to the active site of an enzyme. The calculated result from CASTp showed that the amino acid position 112–235 was predicted to be the active site. Metalloproteases from M4 family need Ca^2+^ and Zn^2+^ as cofactors which bind with specific amino acid residues in enzyme active site for catalysis. In this study, the zinc-binding residues of His-143, His-147, and Glu-167, with Glu-144 having acted as the catalytic residue. Glu144 was responsible for the polarization of the catalytic water molecule leading to an enhancement of nucleophilicity, whereas Asp226 orientated the imidazolium ring of His231 and His231 acted as proton donor and general base [78]. His143 formed a hydrogen bond with Asp171 and had been shown to be essential for activity [79] Tyr158 played a primary role in transition state stabilization and substrate binding [80]. Due to the importance of this active site, it has been widely studied as a target site for antibacterial agents. The 3-D structure of the enzyme is analyzed to identify active site residues as a target site to design drugs which can fit into enzyme.

## Conclusion

Metalloproteases of M4 family are widely dispersed across the nature. The importance of these proteases has been perceived since their roles in bacterial pathogenicity along with in industrial sectors. The present in silico study reveals the bacterial M4 metalloprotease are thermostable, hydrophillic, and extracellular in nature with diverse molecular mass ranging from 38 to 66 KDa. Cross-validation for the 3D model quality assessment was performed by different servers. Hence, this overview may help to get a theoretical idea in developing cross-protective next-generation anti-bacillolysin vaccines as well as to design enzymes with desirable characteristics for biotechnological applications. However, further in vivo studies might be suggested.

## Supplementary Information

**Additional file 1: Supplementary Table.** Details of 31 different M4 metalloprotease from different bacterial sources used in the study.

**Additional file 2: Figure S1.** Multiple sequence alignment of M4 metalloprotease amino acid sequences.

## Data Availability

All the protein sequences are available in Uniprot. Uniprot ID was provided into the manuscript.

## Abbreviations

AI: Aliphatic index
*Ala*: *Alanine*
*Arg*: *Arginine*
*Asn*: *Asparagine*
*Asp*: *Aspartic acid*
*BLAST*: *Basic local alignment search tool*
*CASTp*: *Computed atlas of surface topography of proteins*
EC: Extinction coefficient
Glu: Glutamic acid
*Gly*: *Glycine*
*GRAVY*: *Grand average hydropathicity*
His: Histidine
Leu: Leucine
Lys: Lysine
*MEGA*: *Molecular evolutionary genetics analysis*
*NCBI*: *National center for biotechnology information*
*NPS@*: *Network protein sequence analysis*
*PDB*: *Protein data bank*
pI: Isoelectric *p*oint
PPI: Protein-protein interaction
*Pro*: *Proline*
QMEAN: Qualitative model energy analysis
*Ser*: *Serine*
*SOPMA*: *Self-optimized prediction method with alignment*
*STRING*: *Search tool for the retrieval of interacting genes/proteins*
*Thr*: *Threonine*
*Tyr*: *Tyrosine*
*Val*: *Valine*
*UniProt*: *Universal protein*
