# Supplementary material for: In silico characterization and structural modeling of bacterial metalloprotease of family M4
Source: J Genet Eng Biotechnol. 2021 Feb 2;19:25. doi: 10.1186/s43141-020-00105-y (PMC7851659; doi:10.1186/s43141-020-00105-y)
Supplement: Supplementary file 1 — Additional file 1: Supplementary Table. Details of 31 different M4 metalloprotease from different bacterial sources used in the study. [file 43141_2020_105_MOESM1_ESM.docx]

Supplementary Table: Details of 31 different M4 metalloprotease from different bacterial sources used in the study.

| Serial No | Bacteria Name | MEROPS Family | Protein name | Gene | UniProtKB | Sequence Length |
| --- | --- | --- | --- | --- | --- | --- |
| 1 | *Aeromonas hydrophila* | M4 | Metalloprotease | *N/A* | I2A620 | 590 |
| 2 | *Bacillus thermoproteolyticus* | M4 | Thermolycin | npr | P00800 | 548 |
| 3 | *Bacillus stearothermophilus* | M4 | Thermolycin | nprS | P43133 | 551 |
| 4 | *Bacillus subtilis subsp. Amylosacchariticus* | M4 | Bacillolysin | nprE | P68735 | 521 |
| 5 | *Bacillus subtilis* | M4 | Bacillolysin | nprE | P68736 | 521 |
| 6 | *Bacillus cereus* | M4 | Bacillolysin | npr | P05806 | 566 |
| 7 | *Bacillus brevis* | M4 | Bacillolysin | npr | P43263 | 527 |
| 8 | *Bacillus polymyxa* | M4 | Bacillolysin | npr | P29148 | 590 |
| 9 | *Bacillus caldolyticus* | M4 | Thermolycin | npr | Q59193 | 546 |
| 10 | *Bacillus megaterium* | M4 | Bacillolysin | nprM | P0CH29 | 562 |
| 11 | *Bacillus amyloliquefaciens* | M4 | Bacillolysin | npr | P06832 | 521 |
| 12 | *Bacillus acidocaldarius* | M4 | Thermolysin | *N/A* | Q43880 | 546 |
| 13 | *Bacillus sp. EA1* | M4 | Thermolysin | npr | Q59223 | 546 |
| 14 | *Clostridium putrefaciens* | M4 | λ-toxin | nprM | A0A381J9B8 | 553 |
| 15 | *Enterococcus faecalis* | M4 | Gelatinase | gelE | Q833V7 | 510 |
| 16 | *Erwinia_carotovora* | M4 | Extracellular metalloprotease | prt1 | Q99132 | 347 |
| 17 | *Legionella longbeachae* | M4 | Zinc metalloproteinase MspA | mspA | P55110 | 529 |
| 18 | *Legionella pneumophila* | M4 | Msp peptidase | *N/A* | P21347 | 543 |
| 19 | *Listeria monocytogenes* | M4 | Zinc metalloproteinase | mpl | P23224 | 510 |
| 20 | *Pseudomonas aeruginosa* | M4 | Elastase | lasB | P14756 | 498 |
| 21 | *Renibacterium salmoninarum* | M4 | Zinc metalloproteinase | hly | P55111 | 548 |
| 22 | *Staphylococcus aureus* | M4 | aureolysin | aur | P81177 | 509 |
| 23 | *Staphylococcus epidermidis* | M4 | elastase | sepA | P0C0Q3 | 507 |
| 24 | *Streptomyces lividans* | M4 | Metalloproteinase | SLIV_25315 | D6EEH2 | 547 |
| 25 | *Serratia marcescens* | M4 | Extracellular minor metalloprotease | smp | Q06517 | 352 |
| 26 | *Vibrio proteolyticus* | M4 | vibriolysin | nprV | Q00971 | 609 |
| 27 | *Vibrio anguillarum* | M4 | Virulence metalloprotease | empA | P43147 | 611 |
| 28 | *Vibrio aestuarianus* | M4 | Metalloprotease | VAM | A8JNY9 | 611 |
| 29 | *Vibrio vulnificus* | M4 | Vibriolysin | *N/A* | O06694 | 606 |
| 30 | *Vibrio cholerae* | M4 | Hemagglutinin | hap | P24153 | 609 |
| 31 | *Vibrio splendidus* | M4 | vimelysin | Npr-1 | Q76LC2 | 607 |
